# Long-Term Symptoms among COVID-19 Survivors in Prospective Cohort Study, Brazil

**DOI:** 10.3201/eid2803.212020

**Published:** 2022-03

**Authors:** Lívia P. Bonifácio, Viviane N.F. Csizmar, Francisco Barbosa-Júnior, Ana P.S. Pereira, Marcel Koenigkam-Santos, Danilo T. Wada, Gilberto G. Gaspar, Felipe S. Carvalho, Valdes R. Bollela, Rodrigo C. Santana, João P. Souza, Fernando Bellissimo-Rodrigues

**Affiliations:** Ribeirão Preto Medical School Social Medicine Department, University of São Paulo, São Paulo, Brazil (L.P. Bonifácio, V.N.F. Csizmar, F. Barbosa-Júnior, A.P.S. Pereira, J.P. Souza, F. Bellissimo-Rodrigues);; Ribeirão Preto Medical School Department of Medical Images, Hematology and Clinical Oncology, University of São Paulo, Ribeirão Preto, Brazil (M. Koenigkam-Santos, D.T. Wada);; Ribeirão Preto Medical School Infectious and Tropical Diseases Division of Internal Medicine Department, University of São Paulo, Ribeirão Preto (G.G. Gaspar, F.S. Carvalho, V.R. Bollela, R.C. Santana)

**Keywords:** COVID-19, SARS-CoV-2, post-COVID condition, post-acute COVID-19 syndrome, coronavirus disease, severe acute respiratory syndrome coronavirus 2, viruses, respiratory infections, zoonoses, long COVID, cohort, Brazil

## Abstract

We conducted a prospective cohort study in a population with diverse ethnic backgrounds from Brazil to assess clinically meaningful symptoms after surviving coronavirus disease. For most of the 175 patients in the study, clinically meaningful symptoms, including fatigue, dyspnea, cough, headache, and muscle weakness, persisted for >120 days after disease onset.

Understanding is growing that coronavirus disease (COVID-19) can evolve and continue to cause prolonged symptoms, characterizing the post–COVID-19 condition (*1*–*3*). Potential implications go beyond effects on individual patients and might represent an additional burden on healthcare services and social security, which are both already affected by the pandemic. Therefore, learning more about the long-term repercussions of the disease among different populations is essential. This study aimed to describe the occurrence of long-term physical, psychological, and social consequences among patients who survived COVID-19 and received follow-up care at a post–COVID-19 outpatient clinic at a university hospital in Brazil.

## The Study

This prospective cohort study (RECOVIDA) was performed among patients attending a post–﻿COVID-19 ﻿outpatient clinic at Ribeirão Preto Medical School University Hospital, Ribeirão Preto, Brazil (*4*). The institutional review board approved the research protocol.

All adults with PCR-confirmed COVID-19 with symptom onset during February 1–December 31, 2020, who attended follow-up appointments at the study clinic were eligible. Most participants (85.7%) had been discharged after being hospitalized for COVID-19. The remaining participants (14.3%) were mostly healthcare workers from the study facility. No participants had been previously vaccinated against COVID-19. Patients were classified into 3 groups according to the World Health Organization (WHO) severity classification of COVID-19: mild/moderate, severe, and critical (*5*) (Appendix Table 1).

**Table 1:** Baseline clinical and demographic characteristics among 175 patients surviving the acute phase of COVID-19, Ribeirão Preto, Brazil*

Characteristic	COVID-19 severity			
	Mild/moderate, n = 35 (20%)	Severe, n = 80 (45.7%)	Critical, n = 60 (34.3%)	Total, n = 175
Sex				
M	7 (20)	36 (45)	42 (70)	85 (48.6)
F	28 (80)	44 (55)	18 (30)	90 (51.4)
Mean age, y (SD)	44.9 (+10.3)	57.1 (+15.3)	54.2 (+13.2)	53.7 (+14.4)
Ethnic background†				
White (Caucasian or Latin)	19 (54.3)	36 (45)	25 (41.7)	80 (45.7)
Afro-American (Brown)	10 (28.6)	34 (42.5)	26 (43.3)	70 (40)
Afro-American (Black)	6 (17.1)	8 (10)	6 (10)	20 (11.4)
Asiatic	0	1 (1.3)	2 (3.3)	3 (1.7)
Brazilian Indigenous	0	1 (1.3)	1 (1.7)	2 (1.1)
Mean years of schooling (SD)	13.4 (+5.7)	8.1 (+5.5)	8.3 (+5.4)	9.2 (+5.9)
Mean income/person, USD (SD)‡	407.33 (+313.60)	273.01 (+295,85)	229.33 (+210.40)	285.57 (+279.56)
Median	364.01	200.21‡	182.01‡	216.77‡
Currently works as a health professional				
Yes	23 (65.7)	8 (10)	2 (3.3)	33 (18.9)
No	12 (34.3)	72 (90)	58 (96.7)	142 (81.1)
Mean BMI (SD)§	31.8 (+7.5)	32.1 (+7.3)§	31.1 (+7.5)	31.7 (+7.3)§
BMI >30§	17 (48.6)	44 (56.4)§	23 (38.3)	84 (48.6)§
Underlying conditions				
None	16 (45.7)	16 (20)	10 (16.7)	42 (24.0)
Hypertension	9 (25.7)	35 (43.8)	21 (35)	65 (37.1)
Diabetes	1 (2.9)	26 (32.5)	22 (36.7)	49 (28.0)
Dyslipidemia	2 (5.7)	12 (15)	12 (20)	26 (14.8)
Heart problems (other than hypertension)	1 (2.9)	10 (12.5)	8 (13.3)	19 (10.9)
Rhinitis or sinusitis	3 (8.6)	7 (8.8)	7 (11.7)	17 (9.7)
Cancer	1 (2.9)	9 (11.3)	1 (1.7)	11 (6.3)
Thyroid problems	0	4 (5)	6 (10)	10 (5.7)
Depression or anxiety	1 (2.9)	6 (7.5)	3 (5)	10 (5.7)
Smoking				
Current	0 (0)	2 (2.5)	0	2 (1.1)
Previous	2 (5.71)	18 (22.5)	19 (31.7)	39 (22.3)
Hospitalization				
Yes	10 (28.6)	80 (100)	60 (100)	150 (85.7)
No	25 (71.4)	0	0	25 (14.3)
Mean duration of hospitalization, d (SD)	5 (+4)	9.9 (+5.2)	24.1 (+11.1)	15.3 (+10.9)
Median	4	9	20.5	12

*Values are no. (%) except as indicated. BMI, body mass index; COVID-19, coronavirus disease. †Ethnic background information was self-reported and consisted of Latin American, Caucasian, Afro-American, Asian, and Brazilian indigenous persons. ‡$1 US = R $5,49. Data on financial income by person were missing for 3 participants. §BMI data were missing for 2 participants.

This study was exploratory, and sample size was established through convenience. We aimed to include all patients who attended the clinic during the study period and agreed to participate.

Participants were recruited just before the scheduled medical consultation. After the informed consent form was signed, we performed a structured interview and a brief physical examination. We obtained secondary data from patients’ electronic health records. Laboratory and imaging tests were performed at the attending physician’s clinical discretion. We collected study data by using the Research Electronic Data Capture platform (*6*).

We collected information on economic and demographic social profile, medical history, date of symptom onset, hospitalization data, laboratory and imaging test results, persistent symptoms, and quality of life. We assessed quality of life by using the WHO Quality of Life questionnaire (*7*–*9*) (Appendix). The date of symptom onset was used as the reference for follow-up.

We performed statistical procedures by using Minitab 19.2 (https://www.minitab.com) and Stata version 9 (https://www.stata.com). We used odds ratios, 95% CIs, and Fisher exact tests to verify the association between the persistence of symptoms and the severity of disease.

During the study period, 297 patients had a follow-up medical consultation scheduled at the outpatient clinic. We included 175 patients in this study (Table 1; Figure). In this sample, 20% of participants had illness that was considered mild/moderate, 45.7% were severe, and 34.3% were critical.

**Figure:**
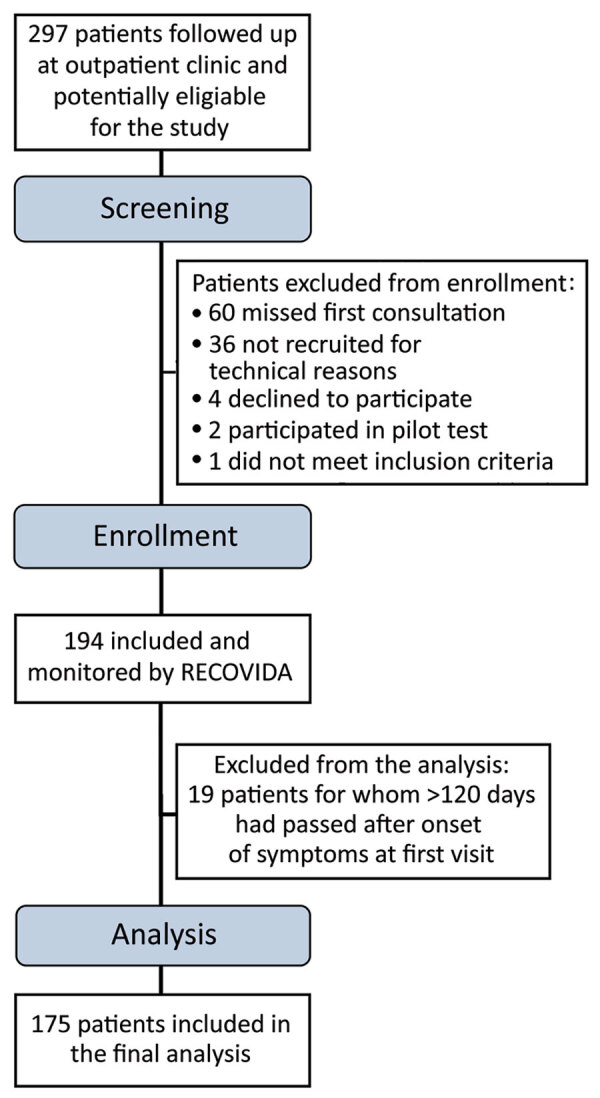
Flowchart of screening and inclusion of coronavirus disease survivors with long-term symptoms in prospective cohort study, Ribeirão Preto, Brazil.

After COVID-19, 80% of the patients experienced persistent symptoms; the 5 most prevalent were fatigue, dyspnea, cough, headache, and loss of overall muscle strength. Compared with the mild/moderate group, patients from the critical group more frequently experienced headaches, change in skin sensitivity, hypogeusia, hyposmia, and loss of muscle strength (Table 2).

**Table 2:** Comparison of long-term symptom occurrence among COVID-19 survivors according to disease severity, Ribeirão Preto, Brazil*

Long-term symptoms	COVID-19 severity									Total, n = 175
	Mild/moderate vs. severe					Mild/moderate vs. critical				
	Mild/moderate, n = 35	Severe, n = 80	OR (95% CI)	p value†		Mild/moderate, n = 35	Critical, n = 60	OR (95% CI)	p value†	
Mean time from onset of symptoms to inclusion, d (SD)	51 (+27)	48 (+23)				51 (+27)	72 (+26)			57 (+27)
Median	43	41				43	74.5			50
Need for readmission	0	1 (1.3)	–	1.000		0	1 (1.7)	–	1.000	2 (1.1)
Persistent symptoms										
No	10 (28.6)	21 (26.3)				10 (28.6)	4 (6.7)			35 (20)
Yes	25 (71.4)	59 (73.8)	1.12 (0.46–2.73)	0.822		25 (71.4)	56 (93.3)	5.60 (1.60–19.58)	0.006‡	140 (80)
Respiratory symptoms	n = 25	n = 59				25	n = 56			140
Dyspnea	9 (36)	22 (37)	1.06 (0.40–2.80)	1.000		9 (36)	15 (27)	0.65 (0.24–1.78)	0.437	46 (32)
Cough	4 (16)	20 (34)	2.69 (0.81–8.92)	0.118		4 (16)	16 (29)	2.10 (0.62–7.09)	0.275	40 (29)
Rhinorrhea	0	4 (7)	–	0.313		0	3 (5)	–	0.549	7 (5)
MRC Dyspnea Scale	n = 9	n = 22				n = 9	n = 15			46
Level 1–2	6 (67)	14 (64)	0.88 (0.17–4.49)	1.000		6 (67)	6 (40)	0.33 (0.06–1.88)	0.615	26 (56)
Level 3–5	3 (33)	8 (36)	0.29 (0.06–1.47)			3 (33)	9 (60)	3.00 (0.53–16.9)	0.412	20 (44)
Cardiovascular symptoms	n = 25	n = 59				n = 25	n = 56			140
Swelling	2 (8)	6 (67)	1.03 (0.16–6.62)	1.000		2 (8)	5 (9)	1.13 (0.20–6.25)	1.000	13 (9)
Hypertension	1 (4)	0	–	0.298		1 (4)	1 (2)	0.44 (0.03–7.27)	0.525	2 (1)
Hypotension	0	1 (2)	–	1.000		0	1 (2)	–	1.000	2 (1)
Neurologic symptoms	n = 25	n = 59				n = 25	n = 56			140
Headache	10 (40)	16 (27)	0.56 (0.21–1.49)	0.304		10 (40)	10 (18)	0.33 (0.11–0.93)	0.050‡	36 (26)
Altered skin sensitivity	1 (4)	4 (7)	1.75 (0.19–16.45)	1.000		1 (4)	21 (38)	14.4 (1.8–114.3)	0.001‡	26 (19)
Hypogeusia	6 (24)	10 (17)	0.65 (0.21–2.03)	0.545		6 (24)	3 (5)	0.18 (0.04–0.79)	0.022‡	19 (14)
Hyposmia	7 (28)	7 (12)	0.35 (0.11–1.12)	0.107		7 (28)	4 (7)	0.20 (0.05–0.76)	0.030‡	18 (13)
Dysgeusia	3 (12)	3 (5)	0.39 (0.07–2.10)	0.356		3 (12)	7 (13)	1.05 (0.25–4.44)	1.000	13 (9)
Loss of recent memory (brain fog)	2 (8)	3 (5)	0.62 (0.10–3.93)	0.631		2 (8)	3 (5)	0.65 (0.10–4.16)	0.642	8 (6)
Gastrointestinal symptoms	n = 25	n = 59				n = 25	n = 56			140
Diarrhea	0	2 (3)	–	1.000		0	4 (7)	–	0.306	6 (4)
Stomach pain	2 (8)	0	–	0.086		2 (8)	0	–	0.093	2 (1)
Other symptoms, n	n = 25	n = 59				n = 25	n = 56			140
Fatigue	10 (40)	23 (39)	0.96 (0.37–2.49)	1.000		10 (40)	22 (39)	0.97 (0.37–2.54)	1.000	54 (39)
Muscle weakness	3 (12)	11 (19)	1.68 (0.43–6.63)	0.539		3 (12)	21 (38)	4.4 (1.17–16.5)	0.033‡	35 (25)
Vision impairment	1 (4)	1 (2)	0.41 (0.03–6.89)	0.509		1 (4)	8 (14)	4 (0.47–33.86)	0.262	10 (7)
Loss of hair	2 (8)	3 (5)	0.62 (0.10–3.93)	0.631		2 (8)	4 (7)	0.89 (0.15–5.18)	1.000	9 (6)

*Values are no. (%) except as indicated. –, calculation could not be performed. †p values were calculated using Fisher exact test. ‡p<0.05.

Regarding quality of life after COVID-19, physical health was more severely affected than the other 3 domains evaluated by the WHO Quality of Life questionnaire (psychological, social relationships, and environmental). Moreover, the comparative evaluation before and after COVID-19 showed a decrease from 81.1% to 68.4% in the percentage of patients who believed that their quality of life was good or very good and an increase from 2.3% to 6.4% of those who believed that their quality of life was poor or very poor. Despite these changes, more than half of patients (56.7%) were satisfied with their current health status at the time of evaluation (Appendix).

## Conclusions

We describe the long-term repercussions of COVID-19 among a sample of patients in Brazil from diverse social and ethnic backgrounds who survived acute infection and attended a follow-up ambulatory clinic appointment. We identified that most patients experienced >1 symptom for >120 days after the onset of disease. This finding also applies to patients who had a mild or moderate form of COVID-19. These symptoms negatively affected the patients’ quality of life; fatigue was the most common symptom, followed by dyspnea and cough.

The clinical picture we describe here, in a population with a mixed ethnic background consisting of Latin American, Caucasian, Afro-American, Asian, and Brazilian indigenous persons, is similar to those encountered in other parts of the world, mainly in Caucasian or Asian populations (*1*,*10*–*12*). Some persistent symptoms found in our study, such as altered skin sensitivity and muscle weakness, primarily affected the patients whose illness was critical, and this finding could be more related to their stay in the intensive care unit than to the COVID-19 itself (*13*).

Several possible pathophysiological explanations for the persistence of symptoms after COVID-19 have been proposed. The most commonly elicited in the literature are direct viral toxicity, endothelial damage, dysregulated immune response, hyperinflammation, hypercoagulability, and poor adaptation of the angiotensin-converting enzyme 2. So far, the actual mechanisms behind this scenario are not entirely understood and deserve further evaluation (*1*,*10*–*13*). Our sample identified that respiratory and heart rates were significantly higher in the patients whose illness was critical, possibly indicating impairment of autonomic function in these patients (*14*,*15*).

We highlight the need to study the persistent symptoms of patients with COVID-19, given the implications for the healthcare system and social security, both of which are already profoundly affected by the pandemic itself. From this perspective, most persons with COVID-19 requiring medical consultation would not be expected to recover fully or resume working immediately after the end of the disease’s acute phase. Instead, they will require a prolonged interdisciplinary healthcare approach focused on physical, mental, and social rehabilitation (*1*,*10*–*15*).

We did not perform genetic sequencing of the severe acute respiratory syndrome coronavirus 2 detected in our patients. Therefore, we cannot evaluate whether different virus variants might affect the occurrence of long-term symptoms among survivors differently.

One of the strengths of our study was our systematic follow-up on participants with prespecified instruments, which ensured high-quality and consistent data. A novelty of the study was that we were able to recruit patients who had mild or moderate COVID-19, which is less common in other studies.

A limitation of our study was the small sample size; the results therefore cannot be generalized to the wider population. Another limitation is the lack of a control group for comparison and selection bias. Most likely, many patients who did not attend a medical consultation after being discharged from the hospital experienced only mild or no prolonged symptoms at all. The same can be said for healthcare workers who were affected by COVID-19 but did not seek medical consultation. The actual prevalence of long-term symptoms among the reference population is unknown, and our data probably overestimate that prevalence.

In summary, it is likely that a substantial proportion of patients surviving COVID-19 will experience long-term symptoms requiring prolonged care, even after mild to moderate disease. These symptoms might negatively affect patients’ quality of life and represent an additional burden for healthcare services and social security.

Appendix Additional information about long-term symptoms among COVID-19 survivors in prospective cohort study, Brazil.
