# Supplementary material for: Long-Term Symptoms among COVID-19 Survivors in Prospective Cohort Study, Brazil
Source: Emerg Infect Dis. 2022 Mar;28(3):730–3. doi: 10.3201/eid2803.212020 (PMC8888217; doi:10.3201/eid2803.212020)
Supplement: Appendix — Additional information about long-term symptoms among COVID-19 survivors in prospective cohort study, Brazil. [file 21-2020-Techapp-s1.pdf]

Article DOI: https://doi.org/10.3201/eid2803.212020

Long-Term Symptoms among COVID-19 Survivors in Prospective Cohort Study, Brazil

Appendix

Variables of Interest

• Demographic, social, and economic data (age, sex, ethnicity, years of schooling, financial income/person)

• Body mass index (kg/m^2)

• Underlying conditions including chronic heart disease (not hypertension); hypertension; diabetes; chronic lung disease; asthma; tuberculosis; chronic kidney disease; chronic liver disease; chronic neurologic disorder; asplenia; cancer; depression/anxiety; HIV; gastrointestinal disease/gastritis; dyslipidemia; thyroid disease; hearing problem or deficit; vision problem or deficit; stroke; prostatic hyperplasia; transplant; previous surgery; obesity (body mass index >30)

• Smoking history

• Hospitalization data

• Laboratory tests, including hemoglobin, hematocrit, lymphocytes, leukocyte count, platelet count, C-reactive protein, lactate dehydrogenase, aspartate aminotransferase, alanine aminotransferase), D-dimer, urea, and creatinine.

• Imaging exam: computed tomography

The chest computed tomography findings regarding the degree of severity and impairment of the lungs, such as those identifying viral pneumonia, were evaluated through consensus of 2 radiologists. Severity was evaluated according to the recommendations of the French Society of Thoracic Imaging.

Abbreviated Version of the WHO Quality of Life Questionnaire (WHOQOL-Bref)

The WHOQOL Group created the WHO Quality of Life Questionnaire (WHOQOL-Bref) to develop a tool with satisfactory psychometric characteristics to assess quality of life in a shorter time (*1–3*).

This instrument is composed of 26 questions; the first 2 questions comprise a self-assessment of quality of life, while the remaining questions represent the facets of each of the domains evaluated: physical, psychological, social, and environmental relationships. The scores of the domains are calculated by summing the mean scores of "n" questions that make up each domain. The result is multiplied by 4, being represented on a scale from 4 to 20, where a score closer to 20 represents a better and more satisfactory overall quality of life and that of each domain evaluated (*1–3*).

References

1. The WHOQOL Group. Development of the World Health Organization WHOQOL-BREF quality of life assessment. Psychol Med. 1998;28:551–8. PubMed https://doi.org/10.1017/S0033291798006667
2. Fleck MP, Louzada S, Xavier M, Chachamovich E, Vieira G, Santos L, et al. Application of the Portuguese version of the abbreviated instrument of quality of life WHOQOL-bref [in Portuguese]. Rev Saude Publica. 2000;34:178–83. PubMed https://doi.org/10.1590/S0034-89102000000200012
3. Pedroso B, Pilatti LA, Gutierrez GL, Picinin CT. Calculating WHOQOL-BREF scores and descriptive statistics through Microsoft Excel [in Portuguese]. Revista Brasileira de Qualidade de Vida. 2010;2:31–6. https://doi.org/10.3895/S2175-08582010000100004

Appendix Table 1. World Health Organization classification of severity of the presentation of COVID-19*

WHO Clinical Classification	On the basis of available clinical records	On the basis of self-report, if clinical records are not available
Mild	No hypoxia or pneumonia	Did not receive oxygen
Moderate	Clinical signs of nonsevere pneumonia AND SpO_2>90% on room air	Did not receive oxygen
Severe	Adults/adolescents: Clinical signs of severe pneumonia AND SpO_2 $\geq$30 breaths/min; Children: Clinical signs of severe pneumonia AND $\geq$1 of the following: central cyanosis; OR SpO_2<90%; OR severe respiratory distress (e.g., fast breathing, grunting, very severe chest indrawing); OR general danger sign(s) (inability to breastfeed or drink, lethargy or unconsciousness, convulsions)	Received oxygen (or said they needed it, but it was not available)
Critical	ARDS; OR sepsis/septic shock; OR pulmonary embolism, acute coronary syndrome, acute stroke; OR multi-inflammatory syndrome in children and adolescents temporally related to COVID-19	Received invasive ventilation (or max available respiratory support)

*Taken from World Health Organization Global COVID-19 Clinical Platform Case Report Form for Post COVID condition (Post COVID-19 CRF), https://cdn.who.int/media/docs/default-source/3rd-edl-submissions/who_crf_postcovid_feb9_2021.pdf?sfvrsn = 76afd14_1&download = true. ARDS, acute respiratory distress syndrome; COVID-19, coronavirus disease; SpO2, oxygen saturation; WHO, World Health Organization.

Appendix Table 2. Data regarding previous hospital admission among 150 patients surviving the acute phase of COVID-19, Ribeirão Preto, Brazil, 2021

Hospitalization Data	Total, n = 150
Ventilatory support/oxygen therapy	
Yes	139 (92.7)
No	11 (7.3)
Admitted to the ICU	76 (50.6)
Mechanical ventilation	
Yes	57 (38.0)
No	93 (62.0)
Mean duration of intubation, d (SD)	13.5 ($\pm$9)
Median	10
Need for vasoactive drugs/vasopressors	42 (28.0)
Need for hemodialysis	12 (8.0)
Any complication during hospitalization	82 (54.6)
Most common complications during hospitalization	
Acute kidney injury	23 (15.3)
Bacterial pneumonia	20 (13.3)
Thromboembolic phenomena	14 (9.3)
Shock	12 (8.0)
Cardiac arrhythmia	7 (4.6)
Anemia	6 (4.0)
Convulsion	3 (2.0)
Pericarditis/myocarditis	2 (1.3)
CT exam during hospitalization	Total, n = 61
Viral pneumonia on CT	
Consistent	56 (91.8)
Nonsuggestive	2 (3.3)
Indeterminate	3 (4.9)
Severity on CT (SIT)	Total, n = 59†
Absent or minimal (<10%)	3 (5.1)
Moderate (10%–25%)	9 (15.2)
Extensive (25%–50%)	26 (44.1)
Severe (50%–75%)	21 (35.6)
Critical (>75%)	0

*Values are no. (%) except as indicated. CT, computed tomography; ICU, intensive care unit; SIT, French Society of Thoracic Imaging.
†In the 2 cases where CT results were considered nonsuggestive of viral pneumonia, the severity was not evaluated.

Appendix Table 3. Long-term clinical and laboratory parameters of COVID-19 survivors, Ribeirão Preto, Brazil, 2021*

	COVID-19 Severity				
Clinical Parameter	Mild/Moderate, n = 35	Severe, n = 80	Critical, n = 60	p value†	Total, n = 175
Respiratory frequency (n = 174)‡					
Mean	17.3‡	19.2	20		19.1
Min–Max	12–34	10–32	12–32		10–34
Median (IQR)	16.5 (14–18.5)	18 (16–22)	20 (16–23.75)	0.012§	18 (16–22)
Oxygen saturation in ambient air, n = 174‡					
<92%	0/35	1/79‡	0/60		1/174
92%–94%	0/35	5/79	2/60		7/174
>95%	35/35	73/79	58/60		166/174
Median (IQR)	98 (97–99)	98 (96–99)	98 (97–99)	0.088	98 (97–99)
Heart rate, n = 173‡					
Mean	77.8‡	78.2‡	87.7		81.4
Min-max	50–103	50–112	53–117		50–117
Median (IQR)	78.5 (69.5–84.5)	78 (71–85)	87.5 (77.25–98.75)	>0.001§	81 (72–88)
Blood pressure, n = 172‡					
SBP>140 mm Hg	7/33‡	20/79‡	15/60		42/172
DBP>90 mm Hg	9/33	28/79	21/60		58/172
SBP<100 mm H	5/33	13/79	10/60		28/172
DBP<70 mm Hg	9/33	31/79	23/60		63/172
Median SBP (IQR)	120 (110–130)	120 (110–140)	120 (110–137.5)	0.623	120 (110–130)
Median DBP (IQR)	80 (70–90)	80 (70–90)	80 (70–90)	0.943	80 (70–90)
Laboratory tests	Mild/Moderate, n = 19	Severe, n = 56	Critical, n = 37	p value†	Total, n = 112
Hemoglobin (ref: 13.9–17.7 g/dL)					
Median (IQR)	13.7 (12.4–14.3)	13 (12.2–14.2)	12.9 (10.8–13.9)	0.396	13 (12.1–14.1)
Hematocrit (ref: 39.6%–51.8%)					
Median (IQR)	42 (37–43)	40 (37–42)	40 (34–43)	0.668	40 (36–42)
Leukocytes (ref: 3.79–10.33 × 10^3/µL)					
Median (IQR)	6.7 (5–7.8)	6.4 (5.2–8.3)	7.7 (6.3–9.7)	0.065	6.8 (5.5–8.6)
Lymphocytes (ref: 1.07–3.12 × 10^3/µL)					
Median (IQR)	1.8 (1.6–2.6)	1.7 (1.3–2)	2.2 (1.7–2.8)	0.001§	1.8 (1.4–2.4)
Platelets (ref: 166–389 × 10^3/µL)					
Median (IQR)	293 (219–337)	239 (191–332)	291 (233–374)	0.113	268 (202–341)
C-reactive protein (ref: <1.0 mg/dL)					
Median (IQR)	0.4 (0.4–1.6)	0.9 (0.4–2.3)	1.4 (0.4–2.9)	0.128	0.9 (0.4–2.3)
LDH (ref: 120–246 U/L)					
Median (IQR)	185.8 (178–237.8)	231.35 (204.2–270)	252.55 (202.25–310.05)	0.024§	236.8 (195.3–289.9)
AST (ref: <38.0 U/L)					
Median (IQR)	22 (16–32)	24.95 (20–34)	23.8 (18.2–29.9)	0.219	24 (18.5–33)
ALT (ref: 10–49 U/L)					
Median (IQR)	22 (12.5–44.5)	40.2 (25–62)	27.5 (17.95–48)	0.023§	35.7 (21–50.1)
D-dimer (ref: <0.5 UG/ml)					
Median (IQR)	0.37 (0.31–0.8)	0.73 (0.52–0.99)	0.92 (0.44–2.54)	0.021§	0.735 (0.41–1.27)
Urea (ref: 19–49 mg/dL)					
Median (IQR)	30.17 (24.4–37.24)	31.88 (25.47–40)	32.96 (24.8–40.87)	0.696	31.78 (25.02–40.76)
Creatinine (ref: 0.70–1.30 mg/dL)					
Median (IQR)	0.78 (0.73–0.96)	0.87 (0.75–0.97)	0.88 (0.74–0.95)	0.766	0.865 (0.735–0.96)

*ALT, alanine aminotransferase; AST, aspartate aminotransferase; COVID-19, coronavirus disease; DBP, diastolic blood pressure; IQR, interquartile range; LDH, lactate dehydrogenase; SBP, systolic blood pressure.
†P values calculated by using the Kruskal–Wallis test.
†‡Missing value.
§p<0.05.

Appendix Table 4. Results of the WHOQOL questionnaire domains (4–20 pts) in inclusion data of COVID-19 survivors, Ribeirão Preto, Brazil, 2021*

Domains	Mean	SD	Minimum value	Maximum value
Physical domain	12.63	1.86	7.43	17.71
Psychological domain	13.89	1.94	7.33	18.00
Social relationships domain	15.72	2.78	4.00	20.00
Environment domain	14.42	2.17	8.50	19.50
Self-assessment of quality of life	14.49	2.89	4.00	20.00
Total	13.97	1.65	8.62	18.15

*COVID-19, coronavirus disease; WHOQOL, World Health Organization Quality of Life.

Appendix Table 5. Results of WHOQOL questionnaire self-evaluation inclusion data of COVID-19 survivors, Ribeirão Preto, Brazil, 2021

Quality of Life Assessment	Before COVID-19, n = 175	After COVID-19, n = 171
Very poor	1 (0.6)	5 (2.9)
Poor	3 (1.7)	6 (3.5)
Neither poor nor good	29 (16.6)	43 (25.2)
Good	113 (64.5)	96 (56.1)
Very good	29 (16.6)	21 (12.3)
Satisfaction with your health (in the past 15 d)		
Very dissatisfied		4 (2.3)
Dissatisfied		20 (11.7)
Neither satisfied nor dissatisfied		50 (29.3)
Satisfied		78 (45.6)
Very satisfied		19 (11.1)

*Values are no. (%). COVID-19, coronavirus disease; WHOQOL, World Health Organization Quality of Life.
